# Anti-mGluR1 encephalitis: Case illustration and systematic review

**DOI:** 10.3389/fneur.2023.1142160

**Published:** 2023-04-17

**Authors:** Osama Khojah, Seraj Makkawi, Saeed Alghamdi

**Affiliations:** ^1^College of Medicine, King Saud Bin Abdulaziz University for Health Sciences, Jeddah, Saudi Arabia; ^2^King Abdullah International Medical Research Center, Jeddah, Saudi Arabia; ^3^Department of Medicine, Ministry of the National Guard-Health Affairs, Jeddah, Saudi Arabia; ^4^Neuroscience Department, King Faisal Specialist Hospital and Research Center, Jeddah, Saudi Arabia

**Keywords:** autoimmune, cerebellar ataxia, metabotropic glutamate receptor 1, mGluR1, antibodies, metabotropic glutamate receptor 1 (mGluR1) antibodies

## Abstract

**Background:**

The literature for immune-mediated neurological disorders is evolving like no other field of neurological illnesses. Many new antibodies or disorders have been described in the last decade. The cerebellum is a brain structure susceptible to these immune-mediated pathologies, and anti-metabotropic glutamate receptor 1 (mGluR1) antibody has a predilection to the cerebellar tissue. Anti-mGluR1 encephalitis is a rare autoimmune disease affecting the central and peripheral nervous systems, triggering an acute or subacute cerebellar syndrome with varying degrees of severity. Anti-mGluR1 encephalitis is a rare autoimmune disease affecting the central nervous system. We aimed to systematically review reported cases of anti-mGluR1 encephalitis and summarize their clinical presentation, management, outcomes, and case reports.

**Methods:**

A search of the PubMed and Google Scholar databases was conducted and included all cases of anti-mGluR1 encephalitis published in English before October 1, 2022. A comprehensive systematic review was conducted using “metabotropic glutamate receptor type 1,” “mGluR1,” autoantibodies,” “autoantibodies,” “autoimmunity,” and “antibody” as keywords. The risk of bias assessment of the evidence was performed using appropriate tools. The qualitative variables were presented as frequency and percentage.

**Results:**

Including our case, 36 cases of anti-mGluR1 encephalitis (19 males, median age 52.5 years, 11.1% pediatric cases) have been reported. The most common clinical manifestations are ataxia, dysarthria, and nystagmus. Initial imaging was normal in 44.4% of patients; however, 75% of patients showed abnormality later in the disease course. The first-line therapy options include glucocorticoids, intravenous immunoglobulin, and plasma exchange. Rituximab is the most commonly used second-line treatment. Complete remission was achieved in only 22.2% of patients, and 61.8% were disabled by the end of their course.

**Conclusion:**

Anti-mGluR1 encephalitis manifests as symptoms of cerebellar pathology. Although the natural history has not been completely elucidated, early diagnosis with prompt initiation of immunotherapy could be imperative. Any patient suspected to have autoimmune cerebellitis should be tested for the presence of anti-mGluR1 antibody in the serum and cerebrospinal fluid. Escalation to an aggressive therapy approach should be applied in cases that do not respond to first-line therapies, and extended follow-up durations are required in all cases.

## 1. Introduction

Metabotropic glutamate receptors (mGluR) are pre- and postsynaptic receptors found in the central and peripheral nervous systems and extensively expressed in Purkinje cells. These receptors are involved in cerebellar development, synaptic transmission, modulation, plasticity, pain perception, memory, learning, and anxiety (1). In the cerebellum, these G-protein coupled receptors are mainly located postsynaptically. mGluR1 is not only expressed at the dendrites of the Purkinje cells but also in parallel fibers and climbing fiber inputs (2). These receptors are essential for cerebellar motor learning, as activating mGluR1 leads to long-term depression of Purkinje cell-parallel fiber synapses (3). Rarely, mGluR1 is targeted by autoantibodies that cause a subacute form of cerebellitis or encephalitis (2). This antineuronal autoimmune reaction was hypothesized to be paraneoplastic in nature as it was associated with malignancies like lymphomas. However, the majority of cases were not associated with any tumors (4). Detection of the antibodies in the cerebrospinal fluid (CSF) or serum and the presence of clinical symptoms are diagnostic of the disease. Stepwise escalation with immunotherapeutic agents, including high-dose intravenous glucocorticoids, intravenous immunoglobulins (IVIg), and/or plasma exchange (PLEX), is used as a first-line treatment for the disease (5). Early initiation of immunotherapy yields better results and prognosis. If the case is severe or not clinically improving, rituximab, cyclophosphamide, azathioprine, or mycophenolate mofetil is used as second-line therapy (6). In this case illustration and systematic literature review, we report the clinical features, 5-year treatment course, and outcomes of a patient with anti-mGluR1 encephalitis. We also describe the disease course, diagnostic test findings, patient outcomes, and treatment approaches for anti-mGluR1 encephalitis outlined in the literature.

## 2. Materials and methods

### 2.1. Literature review

#### 2.1.1. Search methods

We performed a comprehensive systematic review by searching the PubMed and Google Scholar databases. We used “metabotropic glutamate receptor type 1,” “mGluR1,” autoantibodies,” “autoantibody,” “autoimmunity,” and “antibody” keywords in combination with Boolean operators to ensure the inclusivity of all possible results. The search included all reports published until October 1^st^, 2022. The study followed the preferred reporting items for systematic reviews and meta-analyses (PRISMA) guidelines.

#### 2.1.2. Inclusion and exclusion criteria

All published studies that reported at least one anti-mGluR1 encephalitis patient were included. Only studies published in or translated into English were included. The diagnosis of anti-mGluR1 encephalitis had to be based on clinical findings and the presence of mGluR1 antibodies in the serum or CSF. Patients who fulfilled the following criteria were excluded: (1) presence of anti-mGluR1 antibodies in the serum only; (2) low anti-mGluR1 serum titer; (3) positive antibody testing to another neurological autoimmune disease better explaining the patient symptoms. Patients excluded by these criteria included the one reported by Durovic et al. who described a patient diagnosed with anti-MOG encephalitis and was found to be anti-mGluR1 seropositive (titer of 1:40) (7).

#### 2.1.3. Selection of studies

All authors independently assessed the eligibility of each article from the database search. The eligibility of the articles was determined by screening titles and abstracts and then reviewing the full-text versions of the articles. Titles and abstracts were screened by assessing the type of article and population targeted. For example, screened articles involving non-human subjects were excluded. Furthermore, titles and abstracts reporting at least one patient diagnosed with anti-mGluR1 encephalitis underwent further assessment by reviewing the full-text versions of the articles. All disagreements were resolved by consensus.

#### 2.1.4. Data collection

The data collected from each eligible article included age, sex, presence of prodromal symptoms, associated malignancies, clinical manifestations on the first presentation and their duration, leukocyte count, presence of oligoclonal bands in the CSF, antibody titers or presence in the serum and CSF, brain magnetic resonance imaging (MRI) findings, management for acute presentation, maintenance therapy, remission, relapses, presence of antibodies post-therapy, duration of follow-up in months, and disability. Relapse was defined as the acute appearance of new neurological symptoms and/or the recurrence of old symptoms. The geographical origin of a case was determined based on the location of the center described in the methodology or the location of the authors’ primary affiliation. Remission was subdivided into complete, partial, or no remission. Complete remission was defined as complete or near-complete resolution of all symptoms without associated disability, partial remission was defined as meaningful clinical improvement from the first presentation, and no remission was defined as no clinical improvement or worsening of the clinical status. Disability was defined as a limitation in the patient’s physical ability which may or may not require using medical assistive devices such as a wheelchair on their last visit.

#### 2.1.5. Risk of bias and quality assessment

All authors independently and critically appraised the methodological quality of the studies using a modified version of the framework by Murad et al., (8) which was developed to evaluate the risk of bias in case reports and case series. We adapted this tool to assess the reported patients with anti-mGluR1 encephalitis. Of the eight questions described in the assessment framework, five were deemed compatible with our design and adjusted to fit our population. Each question could be answered with a “Yes” or “No” after critically appraising each study. Studies were appraised based on the following: (1) whether the study was specifically conducted to assess patients with anti-mGluR1 encephalitis; (2) if the treatment for anti-mGluR1 encephalitis patients, such as IVIg, glucocorticoids, PLEX, rituximab, tacrolimus, azathioprine, mycophenolate mofetil, cyclophosphamide, and hydroxychloroquine, was adequately ascertained; (3) if the outcome of anti-mGluR1 encephalitis patients was adequately (clinically and radiologically) ascertained; (4) if anti-mGluR1 encephalitis patients were followed up for long enough to determine outcomes, such as relapses or disability, which was set at 12 months or longer; (5) if the study was described in sufficient detail for replication by another investigator or to allow other investigators to make inferences.

A study was considered high quality if it scored “Yes” in more than three questions, moderate quality if it scored “Yes” in two or three questions, and low quality if it scored “Yes” in one or none of the questions. All disagreements were resolved by consensus (Supplementary Table 1).

### 2.2. Ethical considerations

This study was approved by the Institutional Review Board of King Abdullah International Medical Research Center. Written informed consent was obtained from the patient for the publication of any potentially identifiable images or data included in this article.

## 3. Results

### 3.1. Case illustration

**Figure 1:**
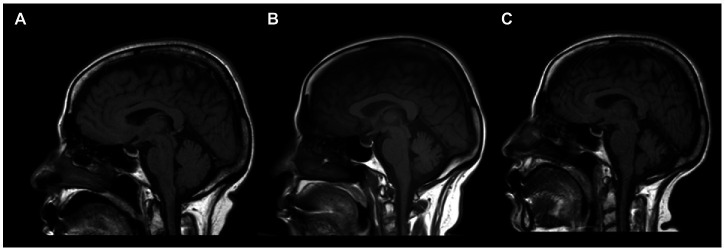
Sagittal brain MRI of an anti-mGluR1 encephalitis patient showing T1 sequence of **(A)** the cerebellar hemisphere upon initial presentation, which later showed progressive cerebellar atrophy at 10 months **(B)** and 20 months **(C)** of follow-up.

A 56-year-old woman presented to a nearby community hospital with slurred speech, unsteady gait, and low-grade fever, which persisted for 2 days. She was admitted with a suspected central nervous system infection and started on antimicrobial therapy. Her medical history was remarkable for type 2 diabetes, hypertension, and osteoarthritis. Computed tomography (CT) findings of the brain were normal. Two days later, the patient was transferred to our hospital (King Abdulaziz Medical City, Jeddah, Saudi Arabia) because her condition did not improve. Upon further questioning, she complained of double vision, fatigue, and generalized body ache. Neurological examination revealed head tremors (titubation), skew deviation, saccadic pursuit with hypometric saccades in horizontal gaze in both directions, and gaze-evoked nystagmus. Hypotonia was noted in both upper and lower limbs. Sensory examination showed reduced sensation in the length-dependent lower extremity. Coordination was impaired with dysmetria in both the upper and lower limbs and severe truncal ataxia with an inability to walk without assistance. Cognitive examination results were normal. CSF analysis showed mild lymphocytic pleocytosis (6 leukocytes/μL) (Normal level: 0–5 leukocytes/μL), a slightly elevated red blood cell count (10 cells/μL) (Normal level: 0 cells /μL), high glucose (6.5 mmol/L) (Normal level: 2.5–4.4 mmol/L), and normal protein (0.42 mg/ml) (Normal level: 0.15–0.6 mg/ml) levels. We made a presumed diagnosis of post-infectious cerebellitis and started the patient on pulse intravenous methylprednisolone (IVMP) (1,000 mg/day) for 3 days, followed by IVIg (1,000 mg/kg) for 5 days. The patient reported mild improvement without functional recovery. CSF cytology and flow cytometry revealed no abnormalities. Serums and CSF samples were sent to Bioscientia International labs in Germany for extensive autoimmune, microbiological, and rheumatological markers analyses. It showed positive oligoclonal bands (OCB) in the CSF, which were absent in the serum, and normal angiotensin-converting enzyme levels in both the CSF and serum. Polymerase chain reaction detected no CSF herpes simplex virus (HSV) DNA types 1 and 2. Results of CSF autoantibody panel that included antibodies against Ca channel (P/Q type), Hu, Ri, Yo, collapsin response mediator protein 5 (CV5/CRMP5), AMPA-1 receptor, metabotropic glutamate receptor 5 (mGlluR5), and metabotropic glutamate receptor 1 (mGluR1) were all negative except for anti-mGluR1 antibodies. CSF and serum anti-mGluR1 antibodies were both detected through indirect immunofluorescence assays with titers of 1:32 and 1:1,000, respectively. Therefore, based on her clinical features and investigational findings, anti-mGluR1 encephalitis was diagnosed. The patient was readmitted for additional immune-modulating therapies and an expedited workup for occult malignancy. Brain MRI showed bilateral, almost symmetrical, subcortical high signal intensity, mostly in the occipital lobes, with no diffusion restriction and no cerebellar signal changes or atrophy. Chest, abdomen, and pelvis CT, mammography and whole-body positron emission tomography (PET)-CT did not show any lesions suspicious of malignancy. Another round of IVMP (1,000 mg/day) and IVIg (1,000 mg/kg) was administered, followed by rituximab (1,000 mg 2 weeks apart then then followed by maintenance of 1,000 mg every 6 months), daily azathioprine (100 mg) and oral steroids upon discharge. Three months later, she was able to walk short distances without assistance, and her slurred speech improved dramatically. After completing 3 doses of rituximab, she was able to function normally at baseline, with mild residual dysarthria and titubation. Two months later, the patient relapsed with a recurrence of disabling ataxia requiring the use of a wheelchair. She was admitted for PLEX and IVMP (1,000 mg/day). The results of repeated CSF analysis remained unchanged and brain MRI showed mild cerebellar atrophy (Figure 1B). After 3 months of biweekly PLEX, remission was achieved again, and the patient could walk unassisted. The patient was then kept on monthly IVIg (1,000 mg/kg). After almost 2 years of monthly IVIg treatment and slow deterioration of her condition, she could not walk without assistance and carry out her activities of daily living. The decision was made to stop IVIg and restart rituximab (1,000 mg) every 6 months. However, due to logistical issues created by the COVID-19 pandemic we were not confident that rituximab infusion will be provided on time, azathioprine (100 mg) was added for about 1 year; once these issues were resolved, we discontinued azathioprine. Six months later, the patient regained functional ability and was able to perform activities of daily living while relying on a walker. A follow-up brain MRI (Figure 1C) showed moderate cerebellar atrophy involving both cerebellar hemispheres and the vermis, with occipital T2 signal changes visualized on her first brain MRI completely resolved. Although repeated CSF analysis showed no signs of inflammation, nor was it positive for OCB, anti-mGluR1 antibodies continued to be present at the same titer. As of now—5 years after the initial presentation—she has mild dysarthria, bilateral dysmetria, and truncal ataxia; her mRS score is 1 and clinical assessment scale in autoimmune encephalitis (CASE) score is 3, repeated cancer screening is still negative, brain MRI is stable and she is on maintenance dose and rituximab (1,000 mg) every 6 months.

### 3.2. Systematic review

**Figure 2:**
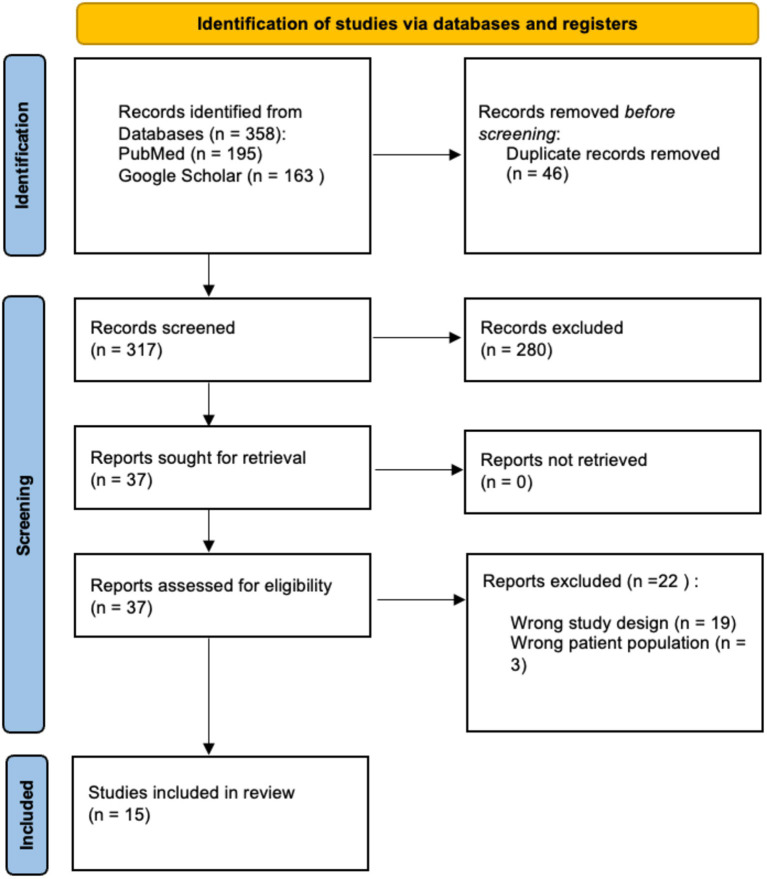
PRISMA 2020 flow diagram for systematic reviews which included searches of databases.

A PRISMA flow diagram describing the case selection process is shown in Figure 2. Fifteen articles (12 case reports and 3 case series) described 35 cases of anti-mGluR1 encephalitis in the literature. Overall, 36 patients were analyzed. The cases originated in the United States of America (9), Spain (10), France (4), Germany (4), Netherlands (3), Italy (2), Brazil (1), Japan (1), Saudi Arabia (1), and Singapore (1).

### 3.3. Literature review

#### 3.3.1. Demographic data and clinical presentations

Overall, 35 cases of anti-mGluR1 encephalitis have been reported in the literature (2, 4, 9–21). Anti-mGluR1 encephalitis affected both sexes equally (1.12:1 M:F). The median age at presentation was 52.5 years (range: 3–81 years). Patients younger than 18 years of age represented 11.1% of all patients and were mostly male (3:1 M:F); 22.2% of all patients had an associated malignancy, six of whom had a lymphoma. Fifty percent of patients with malignancy had it within 5 years of the autoimmune cerebellitis or encephalitis onset; 16.7% (*n* = 6/36) of patients were diagnosed with an autoimmune disease other than that involving mGluR1 antibodies. These autoimmune diseases included multiple sclerosis, Hashimoto’s thyroiditis, Sjögren’s syndrome, and pernicious anemia. Twenty-five percent of patients had one or more prodromal symptoms, including fever, headache, fatigue, weight loss, nausea, vomiting, night sweats, and/or flu-like symptoms; 83.3% of patients had one or more cerebellar symptoms on the first presentation, and later in the disease course, almost all of the patients (94.4%) manifested one or more cerebellar symptoms. Table 1 summarizes the demographic data and clinical features of the previously reported cases. Table 2 describes the symptoms and their proportions during the disease course.

**Table 1:** Demographic features and clinical manifestations on first presentation of 36 patients diagnosed with anti-mGluR1 encephalitis.

Case number	References	Age/gender	Prodromal Symptoms	Associated malignancy	Clinical manifestations on first presentation
1	Sillevis Smitt et al. (11)	19/F	None	Hodgkin’s lymphoma	Cerebellar syndrome
2	Sillevis Smitt et al. (11)	49/F	None	Hodgkin’s lymphoma	Cerebellar syndrome and cognitive decline
3	Marignier et al. (10)	50/F	Yes	None	Cerebellar syndrome and headache
4	Lancaster et al. (9)	69/M	None	None	Cerebellar syndrome
5	Iorio et al. (20)	65/M	None	Mycosis fungoides and prostate adenocarcinoma	Cerebellar syndrome and cognitive decline
6	Lopez-Chiriboga et al. (4)	64/M	None	None	Cerebellar ataxia
7	Lopez-Chiriboga et al. (4)	54/M	None	None	Cerebellar ataxia
8	Lopez-Chiriboga et al. (4)	81/M	None	None	Cerebellar ataxia and cognitive impairment
9	Lopez-Chiriboga et al. (4)	77/M	None	None	Cerebellar ataxia
10	Lopez-Chiriboga et al. (4)	51/M	None	Testicular seminoma	Psychiatric symptoms and dysgeusia
11	Lopez-Chiriboga et al. (4)	60/F	None	None	Dysgeusia
12	Lopez-Chiriboga et al. (4)	58/F	None	None	Cerebellar syndrome and dysgeusia
13	Lopez-Chiriboga et al. (4)	67/M	None	Cutaneous T-cell lymphoma	Cerebellar ataxia
14	Lopez-Chiriboga et al. (4)	67/F	None	None	Paresthesia, vertigo, and dysgeusia
15	Lopez-Chiriboga et al. (4)	33/F	None	Acute lymphocytic leukemia	Cerebellar syndrome and cognitive impairment
16	Lopez-Chiriboga et al. (4)	77/F	None	Mantle cell non-Hodgkin’s lymphoma	Ataxia, spastic paresis, and cognitive impairment
17	Yoshikura et al. (2)	61/F	None	None	Cerebellar syndrome and dysphagia
18	Pedroso et al. (21)	39/F	None	None	Behavioral changes, catatonia, and cerebellar syndrome
19	Christ et al. (12)	45/M	None	None	Dysarthria
20	Gollion et al. (13)	64/M	None	None	Cerebellar ataxia and myoclonic jerks
21	Chaumont et al. (14)	22/F	Yes	None	Cough, headache, and cerebellar syndrome
22	Spatola et al. (15)	29/M	Yes	None	Sleeping difficulties
23	Spatola et al. (15)	22/F	Yes	None	Cerebellar syndrome, fever, hallucinations, and cognitive decline
24	Spatola et al. (15)	45/F	Yes	None	Slowness in writing, hypophonia, and cerebellar syndrome
25	Spatola et al. (15)	59/M	None	None	Cerebellar syndrome
26	Spatola et al. (15)	54/M	Yes	None	Visual loss, cerebellar syndrome, behavioral changes
27	Spatola et al. (15)	56/M	None	None	Behavioral changes and cognitive decline
28	Spatola et al. (15)	62/M	None	Sarcoma	Gait instability
29	Spatola et al. (15)	24/M	None	Hodgkin’s lymphoma	Cerebellar syndrome
30	Spatola et al. (15)	49/F	None	None	Focal seizures and impaired level of consciousness
31	Spatola et al. (15)	6/M	Yes	None	Cerebellar syndrome, tremor and choreiform movements.
32	Bien et al. (16)	3/M	None	None	Unsteady gait
33	Chandler et al. (17)	5/F	Yes	None	Nausea, vomiting, abdominal pain, fever, headache and altered mental status and cerebellar syndrome
34	Vinke et al. (18)	50/F	None	None	Seizures and hallucinations
35	Goh et al. (19)	15/M	None	None	Cerebellar syndrome
36	Current case	56/F	Yes	None	Febrile illness followed by cerebellar syndrome

M, male; F, female.

**Table 2:** Summary of anti-mGluR1 encephalitis symptoms and their proportions.

Symptom	Number of patients	%
**Cerebellar symptoms**	34	94.44
Ataxia	31	86.11
Dysarthria	19	52.78
Nystagmus	10	27.78
Titubation	7	19.44
Dysmetria	7	19.44
Vertigo	6	16.67
Diplopia	4	11.11
Intention tremor	4	11.11
Oscillopsia	2	5.56
**Extra-cerebellar neurological symptoms**	16	44.4
Dysgeusia	4	11.11
Motor changes	3	8.33
Seizures	3	8.33
Myoclonic jerks	2	5.56
Sensory changes	2	5.56
Choreiform movement	1	2.78
Dysphagia	1	2.78
Dystonia	1	2.78
Hypophonia	1	2.78
Loss of vision	1	2.78
Slowness in writing	1	2.78
**Behavioral symptoms**	10	29.41
Apathy	6	16.67
Hallucinations	4	11.11
Catatonia	3	8.33
Personality changes	3	8.33
Irritability	2	5.56
Depression	1	2.78
Impulsivity	1	2.78
Loss of initiative	1	2.78
Paranoia	1	2.78
**Cognitive symptoms**	10	29.41
Cognitive impairment	7	19.44
Memory loss	6	16.67
**General symptoms**	9	25
Fever	4	11.11
Headache	4	11.11
Fatigue	2	5.56
Weight loss	2	5.56
Nigh sweat	1	2.78
Sleep difficulties	1	2.78

#### 3.3.2. Investigations

Reported CSF analysis revealed elevated leukocyte counts in 51.7% and OCBs in 47.8% of patients. Anti-mGluR1 antibodies were detected in the serum of 97.1% and CSF of 96.3% of patients. These antibodies reportedly persisted in 77.8% of patients who were tested in either the CSF or the serum. The presence of anti-mGluR1 antibodies was found in the serum and CSF of 61.1% of patients. In one of patients, it was positive in the serum rather than in the CSF; and positive in two of patients in the CSF but not in the serum. Assessment of the presence of these antibodies in both the serum and CSF was not performed in 30.56% of patients. Initial imaging was normal in 44.4% of patients, but follow-up imaging showed one more finding in 75% of patients. Brain MRI findings included cerebral atrophy, enhancing and non-enhancing brain and spinal cord lesions, as well as cerebellar findings, including cerebellar hyperintensity, enhancement of cerebellar leptomeninges, atrophy, or edema. These cerebellar findings were observed in 52.7% of patients and tended to occur in the medial cerebellar hemispheres and the vermis. Imaging results on the first presentation revealed that 14.7% of patients had generalized or focal brain atrophy, and 41.1% of patients had brain atrophy on follow-up MRIs. Table 3 summarizes the investigative findings of patients reported in the literature.

**Table 3:** Summary of the investigative findings of 36 patients diagnosed with anti-mGluR1 encephalitis.

Case number	References	CSF features		Antibodies titer in serum	Antibodies titer in CSF	Brain MRI
		*Leukocytes/μL*	*Oligoclonal Bands*			
1	Sillevis Smitt et al. (11)	28	Negative	1:3200	1:512	Normal
2	Sillevis Smitt et al. (11)	NA	NA	1:3200	Positive	Normal
3	Marignier et al. (10)	190	Negative	1:20000	1:500	Diffuse cerebellar hyperintensity Follow-up showed moderate cerebellar atrophy
4	Lancaster et al. (9)	8	NA	Positive	Positive	Initially normal Follow-up showed cerebellar atrophy
5	Iorio et al. (20)	Normal	NA	Positive	Positive	Mild cerebellar atrophy
6	Lopez-Chiriboga et al. (4)	Normal	Negative	1:960	NA	Normal
7	Lopez-Chiriboga et al. (4)	NA	NA	1:1920	1:256	NA
8	Lopez-Chiriboga et al. (4)	Normal	Negative	1:1920	1:64	Mild global atrophy and hyperintensity in the central superior cerebellum
9	Lopez-Chiriboga et al. (4)	Normal	Negative	1:61440	NA	Cerebral atrophy
10	Lopez-Chiriboga et al. (4)	29	NA	1:7680	NA	Normal
11	Lopez-Chiriboga et al. (4)	Normal	Negative	1:3840	NA	Normal
12	Lopez-Chiriboga et al. (4)	NA	NA	1:480	NA	NA
13	Lopez-Chiriboga et al. (4)	NA	NA	1:1920	NA	NA
14	Lopez-Chiriboga et al. (4)	NA	NA	1:960	NA	Mild cerebral and cerebellar atrophy
15	Lopez-Chiriboga et al. (4)	NA	Positive	1:1000	Negative	Multiple enhancing brains and spinal cord T2 lesions
16	Lopez-Chiriboga et al. (4)	NA	Positive	1:3200	NA	Multiple non-enhancing brains and spinal cord T2 lesions
17	Yoshikura et al. (2)	5	NA	1:3200	Positive	Initially normal Follow-up showed cerebellar atrophy
18	Pedroso et al. (21)	2	NA	1:12	1:512	Initially normal Follow-up showed cerebellar vermal atrophy
19	Christ et al. (12)	7	Negative	1:100	1:32	Hyperintensity in the medial thalamus and pulvinar predominantly on the left and low cerebellar volume
20	Gollion et al. (13)	Normal	Positive	NA	Positive	Normal
21	Chaumont et al. (14)	214	Positive	Positive	Positive	Cerebellar leptomeningeal contrast enhancement
22	Spatola et al. (15)	17	NA	Positive	Positive	Initially normal Follow-up showed cerebellar atrophy
23	Spatola et al. (15)	214	Positive	Positive	Positive	Gd enhancement of cerebellar leptomeninges
24	Spatola et al. (15)	3	Negative	Positive	Positive	Initially normal Follow-up showed cerebellar vermal atrophy
25	Spatola et al. (15)	2	Positive	Positive	Positive	Cerebellar and brain atrophy
26	Spatola et al. (15)	<5	Negative	Positive	Positive	Unspecific Subcortical dot-like lesions; Follow-up showed cerebellar atrophy
27	Spatola et al. (15)	9	Negative	Positive	Positive	Unspecific Subcortical dot-like lesions; Follow-up showed cerebellar atrophy
28	Spatola et al. (15)	27	NA	Positive	NA	Old ischemic lesions
29	Spatola et al. (15)	4	NA	Positive	Positive	Normal
30	Spatola et al. (15)	<5	Positive	Positive	Positive	Hyperintensities in cerebellar vermis and right frontal lobe
31	Spatola et al. (15)	125	Positive	Negative	Positive	Initially normal Follow-up showed bi-hemispheric cerebellar edema
32	Bien et al. (16)	28	Positive	1:20	1:8	Normal
33	Chandler et al. (17)	39	Positive	NA	1:64	Hyperintensity in cerebellar vermis and medial cerebellar hemispheres
34	Vinke et al. (18)	6–10	Positive	Negative	Positive	Vascular damage
35	Goh et al. (19)	Normal	Normal	Positive	Positive	Normal
36	Current case	6	Positive	1:1000	1:32	Initially normal; Follow-up showed cerebellar atrophy

CSF, cerebrospinal fluid; MRI, magnetic resonance imaging; NA, not applicable; Gd, gadolinium

#### 3.3.3. Management and outcomes

First-line therapy options include glucocorticoids, IVIg, and PLEX. One or more of the aforementioned treatments were used in 83.3% of patients. Patients were administered glucocorticoids (66.7%), IVIg (38.9%), and PLEX (13.9%). Second-line therapy was used in 41.7% of patients as follows: rituximab (27.8%), azathioprine and cyclophosphamide (13.9%), mycophenolate mofetil (11.1%), and tacrolimus and hydroxychloroquine (2.8%); 93.3% of patients who received second-line therapy, failed to have complete remission. More than three treatment modalities were used in 36.1% of cases, yet only 15.4% of whom were able to achieve complete remission. Patients had complete, partial, and no remission in 22.2, 55.6, and 19.4% of cases, respectively. Eventually, 61.8% of patients ended up with some dependency or disability, of whom 57.1% required walking aid, and 9.5% required wheelchair support. Further, 22.2% of patients had one or more relapses—all of whom experienced a disability. Most relapsing patients experienced one relapse, though approximately three episodes have been reported. The median follow-up duration was 24 months. All treatment modalities of the reported cases are summarized in Table 4.

**Table 4:** Summary of management of all reported cases of 36 patients with anti-mGluR1 encephalitis.

Case Number	References	Therapy									Remission	Relapses	Antibodies	Duration of follow-up (Month)	Disability
		*Glucocorticoids*	*IVIg*	*PLEX*	*Rituximab*	*Tacrolimus*	*Azathioprine*	*Mycophenolate mofetil*	*Cyclophosphamide*	*Hydroxychloroquine*					
1	Sillevis Smitt et al. (11)	Yes	Yes	Yes	-	-	-	-	-	-	Complete	None	Resolved	7	No
2	Sillevis Smitt et al. (11)	-	-	Yes	-	-	-	-	-	-	No	None	Persistent	24	Yes
3	Marignier et al. (10)	Yes	Yes	-	-	-	-	Yes	-	-	Partial	None	Persistent	40	Yes
4	Lancaster et al. (9)	Yes	-	-	-	-	-	-	-	-	No	None	NA	36	Yes
5	Iorio et al. (20)	Yes	Yes	-	-	-	-	-	-	-	Partial	None	NA	36	No
6	Lopez-Chiriboga et al. (4)	Yes	-	-	Yes	-	-	-	-	-	Partial	One	NA	17	Yes
7	Lopez-Chiriboga et al. (4)	Yes	Yes	-	-	-	-	-	-	-	No	None	NA	9	NA
8	Lopez-Chiriboga et al. (4)	-	Yes	-	-	-	-	-	-	-	Partial	One	NA	24	Yes
9	Lopez-Chiriboga et al. (4)	Yes	Yes	-	-	-	-	-	-	-	Partial	None	NA	27	Yes
10	Lopez-Chiriboga et al. (4)	Yes	Yes	Yes	-	-	-	-	-	-	Partial	None	NA	11	Yes
11	Lopez-Chiriboga et al. (4)	Yes	-	-	-	-	-	-	-	-	Partial	One	NA	168	Yes
12	Lopez-Chiriboga et al. (4)	-	-	-	-	-	-	-	-	-	Partial	None	NA	6	Yes
13	Lopez-Chiriboga et al. (4)	-	-	-	-	-	-	-	-	-	No	None	NA	4	Yes
14	Lopez-Chiriboga et al. (4)	-	-	-	-	-	-	-	-	-	No	None	NA	60	Yes
15	Lopez-Chiriboga et al. (4)	Yes	-	-	-	-	-	-	-	-	Partial	None	NA	6	No
16	Lopez-Chiriboga et al. (4)	-	-	-	Yes	-	-	-	-	-	No	None	NA	4	Yes
17	Yoshikura et al. (2)	Yes	Yes	Yes	Yes	-	Yes	-	-	-	Partial	Three	Persistent	67	Yes
18	Pedroso et al. (21)	Yes	Yes	-	Yes	-	-	-	Yes	-	NA	None	NA	46	NA
19	Christ et al. (12)	Yes	Yes	-	Yes	Yes	-	-	-	-	Partial	One	Persistent	24	Yes
20	Gollion et al. (13)	Yes	Yes	-	-	-	-	-	-	-	Complete	None	Persistent	10	No
21	Chaumont et al. (14)	-	Yes	-	Yes	-	-	-	Yes	-	Partial	None	NA	12	Yes
22	Spatola et al. (15)	Yes	-Yes	-	Yes	-	-	-	Yes	-	No	None	NA	55	Yes
23	Spatola et al. (15)	-	Yes	-	Yes	-	-	-	Yes	-	Partial	None	NA	12	No
24	Spatola et al. (15)	Yes	Yes	-	Yes	-	Yes	-	Yes	-	Partial	None	NA	20	Yes
25	Spatola et al. (15)	-	-	-	-	-	-	-	-	-	No	None	NA	168	Yes
26	Spatola et al. (15)	Yes	-	-	-	-	-	Yes	-	Yes	Complete	One	NA	120	No
27	Spatola et al. (15)	Yes	-	-	-	-	-	Yes	-	-	Partial	None	NA	90	Yes
28	Spatola et al. (15)	Yes	-	-	-	-	-	-	-	-	Complete	None	NA	66	No
29	Spatola et al. (15)	-	-	-	-	-	-	-	-	-	Partial	None	NA	6	Yes
30	Spatola et al. (15)	-	Yes	-	-	-	Yes	-	-	-	Partial	None	NA	84	No
31	Spatola et al. (15)	Yes	Yes	-	-	-	-	-	-	-	Complete	None	NA	2.5	No
32	Bien et al. (16)	Yes	-	-	-	-	-	-	-	-	Complete	None	Persistent	9.5	No
33	Chandler et al. (17)	Yes	Yes	-	-	-	-	-	-	-	Complete	None	NA	17	No
34	Vinke et al. (18)	-	Yes	-	-	-	Yes	Yes	-	-	Partial	Yes*	Persistent	67	No
35	Goh et al. (19)	Yes	-	-	-	-	-	-	-	-	Complete	None	NA	3	No
36	Current case	Yes	Yes	Yes	Yes	-	Yes	-	-	-	Partial	One	Persistent	61	No
															

IVIg, intravenous immunoglobulin; PLEX, plasma exchange; NA, not applicable

* Number of relapses was not mentioned.

## 4. Discussion

Our illustrated case was an adult patient presented with subacute cerebellar syndrome, diagnosed with anti-mGluR1 encephalitis, and requiring multiple treatment modalities. Our systematic review demonstrated that most patients present with symptoms of cerebellar pathology. Hence, it is imperative to consider anti-mGluR1 encephalitis as a part of the differential diagnosis in any patient suspected to have autoimmune cerebellitis. Brain imaging might be normal in approximately half of the patients. Many patients require multiple treatment options and regimens. However, a minority of patients return to their baseline. In 2000, Smitt et al. published the first two cases of anti-mGluR1 encephalitis in which both patients developed cerebellar ataxia (11). Both patients had Hodgkin’s lymphoma, which had been in remission for multiple years. A history of malignancy, in addition to normal brain MRI findings, prompted the authors to analyze serum and CSF samples for the presence of antineuronal antibodies. After injecting these samples into mice, they were able to elicit an immunohistochemical staining pattern in the brains of the mice that had a distribution pattern similar to that of mGluR1. Evidence for the pathogenicity of anti-mGluR1 antibodies was demonstrated when IgG was injected into mice. After less than an hour, the mice began to show symptoms of cerebellar pathology (11). Novel mutations in the GRM1 gene, which encodes for mGluR1, also reportedly caused progressive forms of cerebellar ataxia in five affected families in Italy (22). Tumor tissue samples were obtained from the patients in Smitt et al. reports; however, none of them expressed mGluR1 or a cross-reactive epitope (11). Contrastingly, the fifth reported case was a patient in remission from mycosis fungoides who presented with ataxia and dysarthria. Eighteen months later, the patient was diagnosed with prostate adenocarcinoma, which after further testing, showed rich expression of mGluR1 and reactivity with anti-mGluR antibodies (20). Of the patients reported in the literature, 77.8% did not have an associated malignancy. Hence, it is still unclear whether malignancies play a role in the development of anti-mGluR1 encephalitis, though continuous testing is still of utmost importance (2). First-line screening for malignancies is CT of the chest, abdomen, and pelvis, although negative results prompt further investigations. PET scans play a substantial role in ruling out occult malignancy. The European Federation of Neurological Societies recommends following up a negative CT with fluorodeoxyglucose-PET in cases with a high index of suspicion for paraneoplastic syndrome (23–25). The trigger of this autoimmune reaction in a non-paraneoplastic form is yet to be completely understood; however, it has been noticed that prodromal symptoms, which echo a viral infection, like in our patient, might trigger this reaction (1). Vague symptoms occurring before the onset of neurological symptoms were reported in 25% of patients. The cause of these prodromal symptoms is unknown. However, one case was preceded by herpes zoster infection in the trigeminal nerve a month before the disease onset, another was preceded by streptococcal pharyngitis 2 months prior, and yet another case was found to have evidence of dengue virus infection. These findings suggest a post-infectious element in the occurrence of anti-mGluR1 encephalitis or that infection may trigger its onset (4, 14, 15). Unlike most autoimmune disorders which favor women, coincident autoimmunity in anti-mGluR1 encephalitis patients affected both genders equally (26). The diagnosis of anti-mGluR1 encephalitis has been based on the presence of neurological symptoms that tend to affect the cerebellum and anti-mGluR1 antibodies in the CSF or serum. However, a threshold for antibody titers to make a diagnosis is yet to be established. The presence of anti-mGluR1 antibodies was found in the serum and CSF in more than half of the patients. The presence of these antibodies in the serum alone was not sufficient to diagnose the patient with anti-mGluR1 encephalitis, as shown by Durovic et al. (7). Relying on the presence of anti-mGluR1 antibodies in the serum alone—of asymptomatic patients or patients diagnosed with other neurological autoimmune diseases—complicate the process of diagnosis (7). Durovic et al. reported a case of MOG encephalitis with anti-mGluR1 antibodies in the serum but not in the CSF (7). They deemed a titer of 1:40 to be too low to be clinically relevant, and the lack of typical cerebellar signs and symptoms made the diagnosis of anti-mGluR1 encephalitis unlikely (7). However, titers lower than those reported by Durovic et al. have been described in a case where the patient had a serum titer of 1:20, although that diagnosis was supported by a CSF titer of 1:8 (16). Lopez et al. described a patient who presented with cognitive and cerebellar symptoms and fulfilled the diagnostic criteria for multiple sclerosis, yet had anti-mGluR1 antibodies in the serum but not in the CSF (4). In both cases, it is difficult to be certain whether these pathologies were particularly due to anti-mGluR1 encephalitis or whether the detection of anti-mGluR1 antibodies was an incidental finding in the context of another autoimmune disease (4, 7). The appearance of anti-mGluR1 encephalitis associated with other autoimmune diseases was reported in 16.7% of cases. Smitt et al. found that the anti-mGluR1 titer per unit of IgG was significantly higher in the CSF than in the serum (11). Moreover, Vinke et al. reported a patient with antibodies detected in the CSF but not in the serum. Further, our patient showed positive OCBs in the CSF but not in the serum, providing evidence of intrathecal synthesis of these antibodies (18). Detection of antibodies in the CSF might be more sensitive than in the serum; this has been illustrated in other antibody-mediated autoimmune encephalitides, such as anti-N-methyl-D-aspartate receptor (NMDAR) encephalitis (27). Thus, studies aiming to assess the sensitivity and specificity of serum and CSF antibody testing in patients with anti-mGluR1 encephalitis are necessary. Lack of cerebellar signs and symptoms is rare but insufficient to exclude the diagnosis. Two cases have been described where the patients never developed any cerebellar signs or symptoms (15). mGluR1 is highly expressed in the cerebellum but is also expressed in the limbic system (hippocampus and olfactory bulb), basal ganglia (globus pallidus, ventral pallidum, and substantia nigra), thalamus, lateral septum, superior colliculus, and parts of the posterior region of the tongue (4, 28). Brain MRI findings and symptoms correlating with each of these structures, such as seizures and psychiatric and cognitive impairment in association with the limbic system, have been reported (18). It is possible for patients who initially present without cerebellar findings to develop them later. Six cases have been reported to first have presented without any cerebellar signs or symptoms, only to develop them later. Additionally, patients presenting with cerebellar signs and symptoms tend to develop other non-cerebellar neurological symptoms later (4). Pediatric patients seem to have a family history of autoimmune diseases, acute symptoms, and symptoms akin to those of movement disorders and cerebellar pathologies (15, 17, 29).

Electroencephalography (EEG) was not utilized in most cases; however, Christ et al. recommended its use for diagnostic purposes, especially when imaging and CSF cell counts were normal (12). CSF cell counts and initial MRI findings were normal in approximately half of the patients. However, over time, brain MRI findings were positive in three-quarters of the patients. These changes in the MRI findings from normal to abnormal are due to Purkinje cell degeneration after continuous exposure to antibodies, which should emphasize the importance of early treatment (2). Treatment of anti-mGluR1 encephalitis relies on immunosuppression, similar to other autoimmune encephalitides. Among patients who received any form of treatment, all but one received one or a combination of glucocorticoids, IVIg, and PLEX. Failure of first-line therapy necessitates the utilization of one or more second-line therapies. Multiple treatment options were used due to ineffective therapy, utilizing another form of therapy during relapse, and/or intolerable side effects. Spatola et al. were unable to find any significant correlation between good outcomes (mRS ≤ 2) and immunotherapy (15). A multicenter study including 577 patients diagnosed with anti-NMDAR encephalitis showed that only 27% of patients required second-line therapy (rituximab and/or cyclophosphamide) (24). Contrastingly, almost all patients with anti-mGluR1 encephalitis who received second-line therapy failed to achieve complete remission. Relapses tended to occur shortly after discontinuation of therapy. However, this was not always the case; our patient relapsed 2 months after completing the third dose of rituximab. Fortunately, relapses responded well to the resumption of therapy. Similarly, Christ et al. found that their patient’s dysarthria worsened while the patient was on IVIg (12). Both the case reported by Christ et al. and our patient were started on rituximab therapy after their functional status continued to deteriorate. This decision yielded a dramatic improvement in both patients. Persistence or resolution of these antibodies does not seem to affect outcomes or disability. Hence, the treatment response should follow clinical symptoms rather than antibody titers in the serum or CSF. Compared to other autoimmune encephalitides, such as anti-mGluR5 encephalitis, anti-NMDAR encephalitis, or anti-LGI1 encephalitis, poorer outcomes are observed in patients diagnosed with anti-mGluR1 encephalitis (15, 30, 31). Non-paraneoplastic cases of anti-mGluR1 encephalitis reportedly have poorer responses to immunotherapy and higher numbers of relapses (1).

### 4.1. Limitations

Considering the retrospective nature of the reports included in this review, data retrieval may be incomplete because of the lack of standardization of reporting and testing. Moreover, the generalizability is hindered by the small number of published reports. Language also represents a barrier that has impeded our ability to retrieve and assess publications that were not written in English. Additionally, asserting that certain ataxia was due to cerebellar pathology might not be entirely possible. For example, thalamic lesions can cause cerebellar-like ataxia.

## 5. Conclusion

Anti-mGluR1 encephalitis is an immune disorder that requires early diagnosis and timely initiation of therapy to achieve improved outcomes. Testing for anti-mGluR1 antibodies should be considered for any acute or subacute cerebellar ataxia, especially following a prodrome of febrile illness or associated with malignancy. Escalation to an aggressive therapy approach should be utilized in cases that do not respond to first-line therapies, and extended follow-up durations are required in all cases. More data are required to identify the most appropriate therapeutic plan to resolve clinical manifestations and prevent possible relapses.

## Data availability statement

The raw data supporting the conclusions of this article will be made available by the authors, without undue reservation.

## Ethics statement

The studies involving human participants were reviewed and approved by King Abdullah International Medical Research Center. The patients/participants provided their written informed consent to participate in this study.

## Author contributions

OK: methodology, formal analysis, investigation, data curation, writing—original draft, and writing—review & editing. SM: conceptualization, methodology, investigation, writing—original draft, and writing—review & editing. SA: conceptualization, methodology, investigation, writing—original draft, writing—review & editing, and supervision. All authors contributed to the article and approved the submitted version.

## Conflict of interest

The authors declare that the research was conducted in the absence of any commercial or financial relationships that could be construed as a potential conflict of interest.

## Publisher’s note

All claims expressed in this article are solely those of the authors and do not necessarily represent those of their affiliated organizations, or those of the publisher, the editors and the reviewers. Any product that may be evaluated in this article, or claim that may be made by its manufacturer, is not guaranteed or endorsed by the publisher.

## Supplementary material

The Supplementary material for this article can be found online at: https://www.frontiersin.org/articles/10.3389/fneur.2023.1142160/full#supplementary-material
