# Supplementary material for: Anti-mGluR1 encephalitis: Case illustration and systematic review
Source: Front Neurol. 2023 Apr 17;14:1142160. doi: 10.3389/fneur.2023.1142160 (PMC10149714; doi:10.3389/fneur.2023.1142160)
Supplement: Supplementary file 1 [file Table_1.DOCX]

Supplementary table 1: Quality assessment of Articles Reporting Anti-mGluR1 Encephalitis Patient(s)

| **Study** | **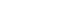**  **Does the patient(s) represent(s) the whole experience of the investigator in the study (was the series specifically related to mGluR1 antibodies)?** | **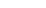**  **Was the exposure (e.g IVIg, Glucocorticoids, PLEX, Rituximab, etc) adequately ascertained?** | **Was the outcome adequately ascertained clinically and radiologically?** | **Was follow-up long enough for outcomes (relapse, disability) to occur (12 months or more)?** | **Is the case(s) described with sufficient details to allow other investigators to replicate the research or to allow practitioners make inferences related to their own practice?** | **Quality** |
| --- | --- | --- | --- | --- | --- | --- |
| Sillevis Smitt et al., 2000 | Yes | Yes | No | No* | Yes | Moderate |
| Marignier et al., 2010 | Yes | Yes | Yes | Yes | Yes | High |
| Lancaster et al., 2011 | No | Yes | No | Yes | Yes | Moderate |
| Iorio et al., 2013 | Yes | Yes | No | Yes | Yes | High |
| Lopez-Chiriboga et al., 2016 | Yes | Yes | No | No** | Yes | High |
| Yoshikura et al., 2018 | Yes | Yes | No | Yes | Yes | High |
| Pedroso et al., 2018 | Yes | No | No | No | No | Low |
| Christ et al., 2019 | Yes | Yes | Yes | Yes | Yes | High |
| Gollion et al., 2019 | Yes | Yes | No | No | Yes | High |
| Chaumont et al., 2019 | Yes | Yes | No | Yes | Yes | high |
| Spatola et at., 2020 | Yes | Yes | No | Yes*** | Yes | High |
| Bien et al., 2020 | Yes | Yes | No | No | Yes | High |
| Chandler et al., 2022 | Yes | Yes | No | Yes | Yes | High |
| Vinke et al., 2022 | Yes | Yes | Yes | Yes | Yes | High |
| Goh et al., 2022 | Yes | No | No | No | No | Low |

Reports with at more than three positive quality assessment criteria were considered low risk of bias (high quality), two or three positive qualities as medium risk (moderate quality), and one or none positive qualities was considered as high risk of bias (low quality).

* One out of two patients was not followed up for the set period.

** Six out of eleven patients were not followed up for the set period.

*** Two out of eleven patients were not followed up for the set period.
